# Head & Face Medicine – a new journal for 'intra-interdisciplinary' science. Why? When? Where?

**DOI:** 10.1186/1746-160X-1-1

**Published:** 2005-08-24

**Authors:** Thomas Stamm

**Affiliations:** 1 Poliklinik für Kieferorthopädie, Universitätsklinikum, Westfälische Wilhelms-Universität, Münster, Germany

## Abstract

The human head and face is the target structure of a large number of medical disciplines which are subject to a continuing trend in medical science – 'ongoing fragmentation' or, to use a better established term, 'opening up new fields'. An adverse side effect of this trend is the separation of scientists, which contributes to a breakdown in communication. Specialization is necessary, but who is able to recombine the pieces of knowledge gained in different branches of science? Who is able to trace back an effect to its cause through the whole system? What is the instrument that enables scientists to think 'laterally', or across disciplines?

To be one of these instruments is the vision of *Head & Face Medicine*. To induce 'intra-interdisciplinary' thinking of scientists by bringing together the findings achieved by different researchers from various specialties, all exploring the same target structure – the human head and face. *Head & Face Medicine's *objective is to support scientists in gaining new insights from different views, to recognize patterns, to extract new thoughts, to recombine them and bring new visions to life.

Evolving tools like the internet, e-publishing, Open Access and open peer review make *Head & Face Medicine *a cross between a traditional journal and a data stream which can be queried, analyzed and processed with the aim of increasing medical knowledge in the area of head and face medicine. These tools represent several advantages: fast publication, increase of a paper's scientific impact and ethical superiority.

*Head & Face Medicine *looks forward to receiving your contributions.

## 

Hardly any region of the human body depends upon the synergism of a variety of medical disciplines to the same extent as the human head. To understand the complexity of the whole system 'head' it is necessary to reduce the system to its most discriminable elements and to explore their nature, because the elements realize certain functions in the whole. This philosophical tradition, the reduction principle, continues to be adhered to and developed in medical science. Attributable to this development is the ongoing fragmentation of medical disciplines into more and more sub-(sub-)specialties; or to put it more positively, one would argue: the opening of new fields.

However, an adverse side effect of this progress is the separation of scientists working in different sub-specialties, resulting in a breakdown in communication. Intensive scientific debate is common within the fields but not across them. Specialization is necessary, but where in this process are the individuals who are able to recombine the pieces of knowledge gained in narrow but deep branches of science? Who is able to trace back an effect to its cause through the whole system? What is the instrument that enables scientists to think lateral or parallel to their own discipline? Definitely, there is not one single instrument, but an important one is communication – communication on different levels of knowledge linked and cross-referenced to disciplines working on the same target structure. The traditional term of this concept is 'interdisciplinary'.

What do we really mean by 'interdisciplinary'? It is not the collaboration with a scientist of an adjacent medical branch who is not interested in your problem. Interdisciplinary is a kind of thinking, and it is initiated in an individual mind. What we really mean is 'intra-interdisciplinary'. To induce this way of thinking we need a particular dose of knowledge from the adjacent disciplines. Therefore, *Head & Face Medicine *has developed a particular vision.

## Head & Face Medicine's vision

Progress in interdisciplinary diagnostics, therapy and research of pathologic conditions of the human head and face by raising new scientific questions which demand new ways of thinking to improve medical quality.

To make this vision come true, we need your help. We need your ideas, insights, observations, and research results, across barriers of specialization, to induce creativity and innovations. This would allow us to learn from all the different disciplines which are involved in head and face disorders, by communicating, by presenting our findings, by defending our hypotheses, and by criticizing ideas and debating methodologies across the frontiers of our own formal training. It would allow us to be interdisciplinary in all ways of thinking to enrich medical knowledge. In the inaugural issue of *Head & Face Medicine*, the co-founders of the journal, Ulrich Meyer and Hans-Peter Wiesmann, will illustrate their vision of 'intra-interdisciplinary', with thoughts from bio-mineralization, tissue-engineering and maxillofacial surgery.

On the way to fulfilling this mission we are grateful that it was possible to establish an international editorial board which reflects the principle of inter-disciplinarity in combination with scientific quality. All members of the editorial board are well known scientists in their respective area of expertise and have agreed to spend a vast amount of their valuable time to support our vision.

Evolution is what we need in scientific literature, not revolution [1]. With BioMed Central we found a publisher who provides a platform where we can use today's 'evolving' tools for scientific literature: e-publication, Open Access and open peer review. Let me convince you by summarizing the advantages of these tools.

## "Publication delay has a harmful effect on patients' health"

It has been emphasized that some of the current processes of publication involve a considerable delay in the dissemination of clinical research, which has a significant effect on patients' health [2]. A review of AIDS trials conducted in 1998 observed a publication delay of between 1.7 and 3 years. Although an improvement, the authors found that in 2004, the publication delay for randomized clinical trials was still 20 month or longer. We agree with the authors that this is unacceptable.

Many solutions have been suggested to promote timely publication. *Head & Face Medicine *aims to provide the authors with a first decision within six weeks after manuscript submission. Immediately on acceptance, the scientific community can read the author's article as a provisional PDF version. Once the journal is included in PubMed (which will occur approximately 2 months after the launch of the journal), the provisional version will be sent to PubMed and included after a 48-hour delay. This will be replaced by the final full-text version when available. *Head & Face Medicine's *general publication process is therefore faster than that of other journals with a 'rapid' publication section [2].

## "Free online availability substantially increases a paper's impact"

Irrespective of economic, moral and ethic arguments on 'Open Access' in scientific literature, what are today's facts? What is the benefit for the individual patient?

It has been shown that Open Access articles were cited 50–300% more often than non-Open Access articles from the same journal and year [3]. The same applies to non-medical science literature, where on average 336% more citations for online articles were observed. There was a clear correlation between the number of times an article is cited and the probability that the article is online [4]. The ability to locate relevant scientific results quickly will dramatically improve scientific progress and therefore improve medical quality. These important findings are cogent and make it imperative for us to turn to Open Access. *Head & Face Medicine *has adopted BioMed Central's Open Access Charter [5], which is the successful base of many independent journals. At this point, it must be clearly stated that no individual who is involved in developing and sustaining *Head & Face Medicine *has competing interests.

### "Most publishing scientists didn't know much about the benefits of Open Access"

About 20% of the total number of articles published annually are Open Access [6] and it has been said that most publishing scientists didn't know much about the benefits of Open Access [3]. Here is a brief description of the Open Access policy of *Head & Face Medicine *and its benefits for science and the general public. For all who want to dive into the Open Access debate we recommend Peter Suber's weblog [7].

All articles of *Head & Face Medicine *become freely and universally accessible online, and so the author's work can be read by anyone at no cost. The authors hold copyright for their work and grant anyone the right to reproduce and disseminate the article, provided that it is correctly cited and no errors are introduced [5]. A copy of the full text of each article is permanently archived in an online repository separate from the journal. *Head & Face Medicine's *articles are archived in PubMed Central [8], the US National Library of Medicine's full-text repository of life science literature, and also in repositories at the University of Potsdam [9] in Germany, at INIST [10] in France and in e-Depot [11], the National Library of the Netherlands' digital archive of all electronic publications.

## Open peer review is superior to traditional closed peer review – ethically

There are many arguments for and against open peer review. At present there is no evidence that any single kind of peer review leads to higher quality reports and feedback. *Head & Face Medicine *uses an open system because the ethical reasons to move away from anonymity are significant. Many publishing scientists have seen brusque, incompetent and destructive reports produced in anonymity. The worst abuses are blocking or stealing of new ideas. This behavior is not acceptable and we strongly believe that transparency leads to a more respectful and constructive communication.

### Head & Face Medicine supports the reviewers' academic credit for the work they do

Reviewing can seem a thankless task in anonymity. In a closed system, reviewers don't receive academic credit for the power and knowledge they invested to improve the quality of an authors work. Therefore, *Head & Face Medicine *posts the signed reviews in a pre-publication history, which is freely available to access from the published article. Reviewers' names and reports are therefore easily accessible via the published article, which leads to wider recognition within the scientific community. With the open system, both sides win. The authors receive a constructive, high-quality contribution with a higher chance of acceptance [12], and the reviewers improve their academic reputation. Then, if a paper is frequently cited, all parties involved have the benefit of the scientific impact.

## Résumé

The vision of *Head & Face Medicine *is to induce 'intra-interdisciplinary' thinking by bringing together the findings of different researchers from various specialties, all exploring the same target structure. The objective is to gain new insights from different views, to recognize patterns, to extract new thoughts, to recombine them and to bring new visions to life. Scientists have the ethical duty to publish their results as soon as possible, irrespective of whether or not the findings are negative, which means challenging current dogmas, tenets or opinions of experts. The internet, e-publishing, Open Access and open peer review turn a journal like *Head & Face Medicine *to a cross between a traditional journal and a data stream which can be queried, analyzed and processed with the aim to improve medical knowledge in the area of head and face medicine. We look forward to receiving your contributions.
